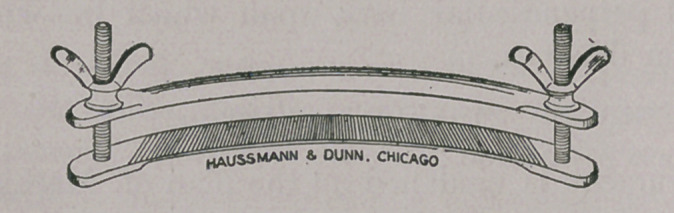# Department of Canine and Feline Medicine and Surgery

**Published:** 1901-03

**Authors:** Cecil French

**Affiliations:** Washington, D. C.


### DEPARTMENT OF CANINE AND FELINE MEDICINE AND SURGERY.

By Cecil French, D.V.S.,
WASHINGTON, D. C.

### SOME NEW CANINE INSTRUMENTS.

Herewith are descriptions and illustrations of a few instruments I have recently devised for canine practice. In bringing them to the notice of the profession I wish to state that I do not claim entire originality in their construction. The old adage "there is nothing new under the sun" is equally applicable to the contrivance of surgical instruments as it is to everything else. I have made use of well-known principles in their construction so that they might be peculiarly serviceable in canine surgery.

### Mouth Speculum.

I believe this will be found to be a very practicable instrument of its kind. It is constructed on the same principle as the Wingrave mouth-gag for human surgery.

In devising a mouth speculum one must keep one essential object in view: as far as possible no parts of the instrument must be allowed to interfere with free access to the entire buccal cavity.

Speculums which consist of spreading horizontal bars supported by perpendicular bars defeat in large measure the object at which they aim. The perpendicular bars, which must necessarily find position immediately posterior to the canine teeth, offer an undesirable obstruction to lateral passage of instruments and fingers of the operator. I have always found this objection a very decided one when operating for ranula, for instance.

In my speculum the perpendicular bar finds position well back of the angle of the mouth, where it is entirely out of the way.

This instrument is very light and strong, can be adjusted to meet the requirements of any sized mouth, and has the great merit of being self-expanding.

The parts consist of two stationary horizontal side-bars on each side, into which two oval, curved perpendicular parallel bars are fastened ; two sliding horizontal side-bars run on the perpendicular ones. They are spread by the action of two spiral springs. Upper and lower cross-bars are attachable to the ends of the side-bars at varying distances, being provided with several holes for this purpose. This permits of adjustment to mouths of any width. Screws provided with loops join the cross-bars and side-bars together. Straps are attached to the screw-loops to keep the speculum in place. Attached to either end of one cross-bar is a swinging D (not shown in the illustration), which engages the other cross-bar to keep the speculum closed while introducing it within the mouth.

The instrument is manipulated as follows : After it has been introduced within the mouth, the cross-bars being placed immediately behind the canine teeth, it is buckled securely to the upper and lower jaws by means of the straps. The swinging Ds are then displaced. This releases the sliding-bars, which thereupon expand automatically. In large animals possessed of much mandibular power it is sometimes necessary to assist in the opening by gently forcing the jaws apart. To remove the instrument from the mouth it is necessary only to unbuckle the straps and dislocate it.

To bring the cross-bars into approximation equal pressure must be exerted both upon them and the far extremities of the sliding horizontal bars, in order to overcome the binding action of the curved, oval perpendicular bars, upon which the whole action of the expansion depends.

### Tail Guillotine.

This instrument is modelled on the plan of Mathieu's tonsillotome of human surgery. It is a very convenient article for tailamputation, particularly of puppies, its action being almost immediate. It is worked by means of the thumb and first two fingers, which pull the knife through the tissues.

### Parturition Hook.

This instrument is very similar to one of English origin, the name of which I cannot call to mind. In the English instrument there is a hook at either end, the one blunt and the other sharp. Such an arrangement effectually prevents the operator from securing a good grasp ; in fact, it is almost impossible to employ any traction with it. Moreover, the sharp hook is unnecessary and at all times a menace to the animal when in use. I have eliminated the sharp hook and provided a cross-bar in its place, which makes it a very serviceable instrument in parturition cases. It is made in two sizes.

### Clamps for Amputation of the Ear-flap.

For those who have to do much cropping this little instrument will be found indispensable. It is made in two styles—one straight (for Great Danes), the other curved (for bull, Boston, and black-and-tan terriers).

It is exceedingly light yet strong, and by virtue of its length can be used on the smallest or largest dog. It is very similar to the scrotal clamp of human surgery.

In using it the thumb-screws are loosened and as much of the ear-flap as it is desired to remove is passed between the two halves. These are tightly approximated by turning the thumb-screws. The occluded portion of ear-flap is quickly severed by running a blade along the edge of the clamp. The clamp may be allowed to remain in place for fifteen or twenty minutes if it is desired to arrest hemorrhage, but the latter always stops in a short time of its own accord.

## Figures and Tables

**Figure f1:**
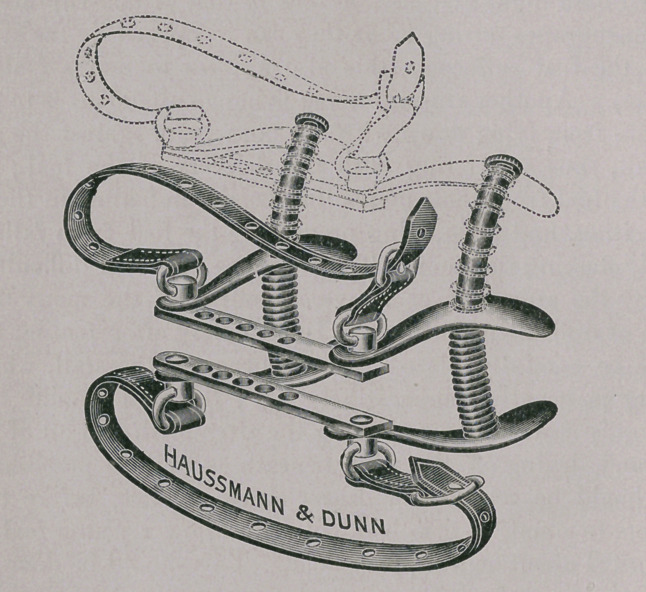


**Figure f2:**
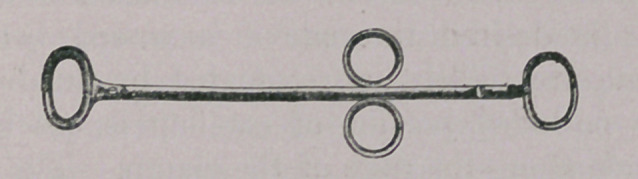


**Figure f3:**
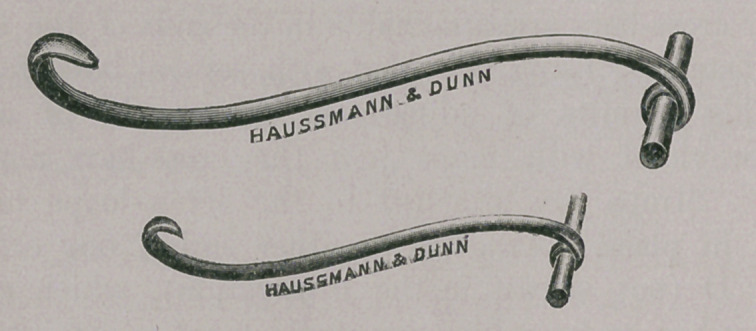


**Figure f4:**